# A comparative study of serum and fecal calprotectin levels in necrotizing enterocolitis

**DOI:** 10.1016/j.jped.2025.101428

**Published:** 2025-08-21

**Authors:** Sara Erol, Cuneyt Tayman, Sabriye Korkut, Ufuk Çakir, Abdullah Kurt, Ismail Koyuncu

**Affiliations:** a Department of Pediatrics, Division of Neonatology, Ankara Bilkent City Hospital, Ankara, Turkey; b Harran University Faculty of Medicine, Medicinal Biochemisty, Sanliutfa, Turkey; c Ankara Yıldırım Beyazıt University, Faculty of Medicine, Ankara, Turkey

**Keywords:** Necrotizing enterocolitis, Prematurity, Calprotectin, Prediction

## Abstract

**Objectives:**

Necrotizing enterocolitis is a significant cause of morbidity and mortality in premature infants. Various fecal, urinary, and serum biomarkers have all been investigated for their potential in the prediction and early detection of necrotizing enterocolitis. This pilot study aimed to explore the feasibility and clinical utility of measuring serum and fecal calprotectin levels in preterm infants with necrotizing enterocolitis.

**Methods:**

This prospective pilot study included preterm infants born at < 32 weeks’ gestation with a birth weight of ≤ 1500 g, consisting of patients diagnosed with necrotizing enterocolitis stage II or III and a randomly selected control group without necrotizing enterocolitis. The relationship between serum and fecal calprotectin concentrations and necrotizing enterocolitis severity, need for surgical intervention, and mortality was systematically analyzed.

**Results:**

A total of 39 necrotizing enterocolitis patients (25 with stage II, 14 with stage III) and 20 randomly selected preterm infants were included as the control group. Serum and fecal calprotectin levels were significantly higher in necrotizing enterocolitis stage III and in infants who required surgery or died (*p* < 0.05), indicating their potential to predict disease severity and poor outcomes.

**Conclusions:**

This pilot study suggests that dual assessment of serum and fecal calprotectin may provide insight into necrotizing enterocolitis severity and outcomes. Larger studies are needed to validate these findings and determine clinical applicability.

**Trial Registration:**

This study was registered with the ClinicalTrials.gov database under the registration number NCT06693154 on November 15, 2024.

## Introduction

Necrotizing enterocolitis (NEC) is a significant cause of morbidity and mortality in premature infants. It occurs in approximately 10 % of preterm infants born weighing <1500 g^1^ with an overall incidence of 5–7 % among all preterm infants.^2^ Despite significant advances in neonatology, there has been no notable reduction in NEC-related mortality rates, with mortality still ranging from 30–50 % in preterm infants born weighing <1000 g.^3^

Although NEC is typically explained by a common pathogenesis characterized by ischemia, inflammation, and bacterial invasion,^4^ there is evidence from studies suggesting that it is not a homogeneous disease but rather a complex and multifaceted disorder. In the prediction of NEC and the differential diagnosis of conditions with clinical presentations similar to classic NEC, such as neonatal sepsis, spontaneous intestinal perforation, ischemic intestinal necrosis, food protein-induced enterocolitis syndrome, and congenital bowel anomalies, a personalized approach is crucial.^5^ At this point, beyond traditional clinical and radiographic findings, specific imaging techniques such as ultrasound, near-infrared spectroscopy for assessing perfusion, and biomarkers can be utilized.^6^

Calprotectin is a cytosolic protein complex from the S100 family, known for its calcium-binding properties.^7^ Comprising S100A8 and S100A9 monomers, calprotectin is expressed variably across different cell types. However, it is consistently present in neutrophils, as well as in monocytes, macrophages, dendritic cells, keratinocytes, and squamous mucosal epithelial cells.^8^ The formation of heterotetramers through the combination of S100A8 and S100A9 heterodimers is crucial for calprotectin's functions both intracellularly and extracellularly.^9^ Calprotectin modulates the immune system through intracellular mechanisms that facilitate the presence of inflammatory cells and mediators, such as arachidonic acid, at sites of inflammation.^10^ It also has extracellular effects by interacting with Toll-like receptor 4 and receptor for advanced glycation end products.^11^

Clinical research has explored the use of calprotectin as a biomarker in intestinal diseases and sepsis, despite its variable levels influenced by clinical factors in neonates. While fecal calprotectin levels have been widely studied in neonatal intestinal diseases, including necrotizing enterocolitis, there is a lack of research on serum calprotectin levels specifically for necrotizing enterocolitis.^12^,^13^ This pilot study aimed to explore the feasibility and clinical utility of measuring serum and fecal calprotectin levels in preterm infants with NEC.

## Material and methods

This prospective pilot study included newborns admitted to the neonatal intensive care unit (NICU) over a 2-year period, born at or before 32 weeks of gestation with a birth weight of ≤ 1500 g, who were included in this study. Ethical approval was obtained from the local ethics committee (number: 26,379,996/26, 03–2022), and informed consent was secured from the parents. This study was registered with the ClinicalTrials.gov database under the registration number NCT06693154 on November 15, 2024.

Data collected included gestational age, birth weight, gender and mode of delivery, as well as the need for resuscitation in the delivery room. Information regarding invasive mechanical ventilation therapy and co-morbidities, including respiratory distress syndrome (RDS), patent ductus arteriosus (PDA), and intracranial hemorrhage, was also recorded. Maternal factors, such as age, maternal infection, and antenatal steroid treatment, were retrieved from obstetric records.

According to the unit’s standardized clinical protocols, early and exclusive human milk feeding is strongly encouraged. Donor milk is not available; therefore, if maternal milk remains insufficient beyond the initial postnatal period, preterm formula is introduced under clinical supervision. Clinical symptoms, including episodes of apnea and desaturation, bradycardia, lethargy, and irregular body temperature, were evaluated alongside gastrointestinal symptoms such as feeding intolerance, vomiting, increased gastric residual volume, bilious or bloody gastric aspirate, decreased bowel sounds, bloody stools, abdominal distension, tenderness, and changes in abdominal skin color. If these symptoms were present, laboratory and radiographic evaluations were conducted with suspicion of NEC. Abdominal radiographs were reviewed by radiologists and neonatologists for abnormal findings, including bowel dilatation, the presence of dilated and fixed bowel loops, bowel wall thickening, ascites, pneumatosis intestinalis, portal venous gas, pneumoperitoneum, and subdiaphragmatic free gas. Laboratory evaluations included blood gas analysis, complete blood count, blood smear, serum electrolytes, blood urea nitrogen, creatinine, liver function tests, and blood cultures.

NEC diagnosis was confirmed based on clinical, laboratory and radiological findings, and the disease was staged into stages I, II, and III using the modified Bell criteria.^14^ Only patients with stage II and stage III were included in the study, while those with stage I (suspected NEC) were excluded. Management was carried out by a dedicated expert team comprising pediatric surgeons and neonatologists, who also made the decisions regarding surgical intervention. Empirical antibiotic therapy with ampicillin and gentamicin is initiated in suspected cases of sepsis or NEC, in line with the institutional antibiotic stewardship guidelines. The duration of antibiotic therapy is determined based on clinical improvement and blood culture results. If the culture is positive, the treatment is adjusted according to the identified microorganism. The criteria for surgical intervention included the presence of necrotic bowel loops identified through serial radiographs showing fixed bowel loops, pneumoperitoneum, and/or persistent metabolic acidosis, shock, or severe thrombocytopenia.

Blood samples for the evaluation of serum C-reactive protein (CRP), interleukin-6 (IL-6), and calprotectin concentrations were collected at the time of NEC diagnosis, whereas fecal samples for calprotectin analysis were obtained on the same day. Blood samples were obtained using Minicollect® 1cc serum tubes (Greiner Bio-One, Kremsmünster, Austria), and fecal samples were immediately stored at −20 °C for batch analysis. All laboratory analyses were conducted by a single individual after the completion of patient recruitment.

The control group consisted of newborns born at or before 32 weeks of gestation, with a birth weight of ≤ 1500 g, who did not develop NEC or show signs of neonatal sepsis. Blood samples were collected from peripheral veins on the third day of life to measure CRP, IL-6, and calprotectin concentration. This timing was chosen to exclude potential inflammation or systemic effects prior to the typical onset of NEC and aligns with routine neonatal intensive care practices, ensuring standardized and systematic sample collection. Fecal calprotectin was not included in the control group analysis, as a substantial proportion of preterm infants had not yet passed meconium on day 3, when serum inflammatory markers were collected. The presence of meconium or the absence of stool at that time point could have introduced considerable variability and compromised the reliability and consistency of early fecal calprotectin measurements.

### Measurement of serum and fecal calprotectin levels

Calprotectin levels were measured using a commercial ELISA kit (Cat. No. RD191217100R, BioVendor Laboratory Medicine, Inc., Karasek 1/1767 Brno, Czech Republic). Serum levels were reported in μg/mL, and fecal levels in μg/g.

### Measurement of CRP and IL-6 concentration

Serum CRP concentration was measured using the nephelometric method (sensitivity = 8 mg/L) (CRP kit, Roche, Germany) on the IMMAGE® system (Beckman-Coulter, USA). IL-6 concentration was measured using a solid-phase enzyme-labelled chemiluminescent immunometric assay (IL-6 kit, Siemens Healthcare Products Ltd., Hanbers, USA) (sensitivity = 2 pg/mL) on the IMMULITE® 2000 system (USA), and the results were recorded.

### Power analysis and sample size considerations

As a pilot study, this investigation aimed to evaluate the feasibility and preliminary utility of serum and fecal calprotectin measurements in preterm infants with NEC. The sample size was limited due to the restricted number of eligible NEC cases meeting the inclusion criteria during the study period. However, a preliminary power analysis was performed to support the methodological validity of the study design. Assuming a power of 80 % and a significance level of 0.05, a minimum of 12 patients per group was estimated to be sufficient to detect a difference of 1 standard deviation (SD) in serum calprotectin levels between NEC and control groups. For fecal calprotectin, which is known to exhibit greater biological variability, an estimated SD of 1.5 was considered in the analysis.

### Statistical analysis

Statistical analysis was conducted using the SPSS 20.0 statistical software package (Chicago, IL, USA). The normal distribution of variables was assessed with the Shapiro-Wilk test, and the results were used for group comparisons. Descriptive statistics were presented as mean and standard deviation or median (25th–75th percentile); categorical variables were presented as count and percentage. For between-group analyses of parametric variables, ANOVA and Chi-square tests were used, while Kruskal-Wallis and Mann-Whitney U tests were employed for non-parametric variables. Bonferroni correction was applied for multiple comparisons. The Chi-square test was used to compare categorical variables in independent groups. The Friedman test and Bonferroni-adjusted Wilcoxon test were used for comparing dependent groups. Correlation analyses were performed using Spearman’s rank correlation, with correlation coefficients (r) and corresponding p-values reported. Receiver operating characteristic (ROC) analysis was performed using the Youden Index to determine the optimal threshold values, and the area under the curve (AUC) was calculated to quantitatively assess the model’s discriminative ability. A p-value of < 0.05 was considered statistically significant.

## Results

Among the 387 preterm infants with a gestational age of <32 weeks and a birth weight of ≤1500 g admitted to the neonatal intensive care unit during the study period, 46 (11.9 %) were diagnosed with NEC, including 25 with stage II and 21 with stage III disease. A total of 39 patients, from whom samples could be sent for serum and fecal calprotectin levels, were included as the study group. Twenty patients who did not develop NEC were randomly chosen from the entire cohort to serve as the control group.

The mean birth weight in the control group was 1125 ± 325 g, compared to 1120 ± 342 g in the NEC group. In the control group, the mean gestational age was 28.3 ± 0.5 weeks, compared to 28.4 ± 0.4 weeks in the NEC group. There were no statistically significant differences between the study and control groups in terms of birth weight, gestational age, maternal age, presence of maternal infection, mode of delivery, need for resuscitation in the delivery room, antenatal steroid administration, additional morbidities associated with prematurity, or the requirement for invasive mechanical ventilation (*p* > 0.05) (Table 1).

**Table 1:** Clinical characteristics of the patients.

Variables	Control group (*n* = 20)	Study group (*n* = 39)	P
Gender (male) n( %)	11(55)	19(48.7)	0.53
Birth weight	1125 ± 325	1120 ± 342	0.67
Gestational age	28.3 ± 0.5	28.4 ± 0.4	0.49
Modes of delivery (C/S) n( %)	12(60)	23(58.9)	0.45
Mothers’ age	28.8 ± 1.2	29.2 ± 0.9	0.25
Maternal infection n( %)	6(30)	12(30.7)	0.76
Antenatal steroid administration n( %)	14(70)	29(74.4)	0.8
Resuscitation in delivery room n( %)	10(50)	22(56)	0.52
Co-morbidities n( %)			
PDA	12(60)	24(61.5)	0.96
RDS	15(75)	30(76.9)	0.64
İCH	7(35)	15(38.5)	0.54
Invasive mechanical ventilation therapy n( %)	12(60)	25(64.1)	0.75

Significant differences in CRP, IL-6, and serum calprotectin concentrations were observed among patients with NEC stage II, NEC stage III, and the control group. Specifically, levels were significantly higher in both NEC groups compared to controls. Additionally, IL-6 and serum calprotectin, but not CRP, were significantly elevated in NEC stage III compared to stage II (Table 2).

**Table 2:** Comparison between control, NEC stage II, and NEC stage III.

Variables	Control group (*n* = 20)	Stage II NEC (*n* = 23)	Stage III NEC (*n* = 16)	P
*IL–6 (pg/mL)	16.8 (6.2–27.4)	66.6 (6.2–126.9)	96 (40–776.1)	<0.001
*CRP (mg/L)	2.9 (1.7–4.1)	27.2 (15.9–38.4)	16.2 (3.3–28.9)	<0.001
⁎⁎Serum calprotectin(μg/mL)	0.79±0.33	28.7 ± 1.85	38.2 ± 9.67	<0.001

⁎ Data are presented as median (25th–75th percentile).

⁎⁎ Data are presented as mean ± standard deviation.

When comparing CRP, IL-6, serum calprotectin, and fecal calprotectin concentration between patients who underwent surgery and those who did not, as well as between NEC survivors and non-survivors, it was found that IL-6, serum calprotectin, and fecal calprotectin concentration were elevated in both infants requiring surgical intervention and those who did not survive (Table 3).

**Table 3:** Comparison between operated and non-operated NEC patients.

Variables	Operated (*n* = 15)	Non-operated (*n* = 24)	p	Survived (*n* = 27)	Deceased (*n* = 12)	p
*IL–6 (pg/mL)	78.4 (49.51–603.61)	56 (21.88–203.38)	**<0.001**	56.0 (17.15–170.15)	78.4 (66.8–710.8)	**<0.001**
*CRP (mg/L)	16.2 (14.9–40.7)	27 (14.2–39.9)	**<0.001**	29 (14.8–43.2)	16.2 (10.5–25.2)	**<0.001**
⁎⁎Serum calprotectin (μg/mL)	39.67±16.21	23.67±9.65	**<0.001**	28.6 ± 8.61	44.9 ± 21.53	**<0.001**
*Fecal calprotectin (μg/g)	36.7 (24.67–68.07)	21.7 (13.9–29.5)	**<0.001**	19.6 (11.25–27.95)	46.4 (29.41–81.5)	**<0.001**

⁎ Data are presented as median (25th–75th percentile).

⁎⁎ Data are presented as mean ± standard deviation.

**Figure 1:**
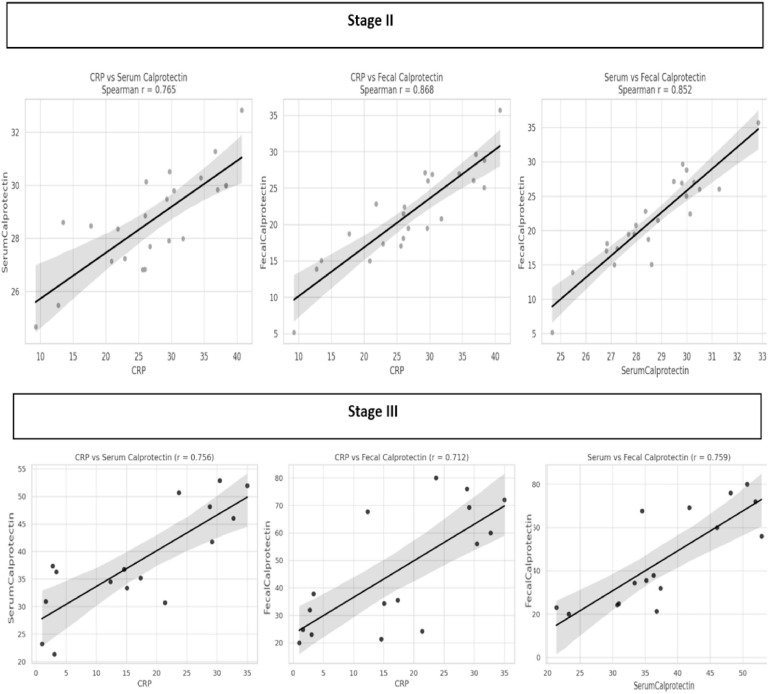
Inflammatory marker correlations in NEC stage II and stage III.

Correlation analyses revealed significant associations between inflammatory biomarkers in both disease stages. In NEC stage II, strong correlations were observed between CRP and serum calprotectin (*r* = 0.765, *p* < 0.05), CRP and fecal calprotectin (*r* = 0.868, *p* < 0.05), and between serum and fecal calprotectin (*r* = 0.852, *p* < 0.05). Similarly, in NEC stage III, CRP was moderately correlated with serum calprotectin (*r* = 0.756, *p* < 0.05) and fecal calprotectin (*r* = 0.712, *p* < 0.05), while serum and fecal calprotectin showed a strong correlation (*r* = 0.759, *p* < 0.05) (Figure 1).

Serum calprotectin demonstrated the strongest predictive performance for surgical intervention (cutoff: 37.84 μg/mL; sensitivity: 88.9 %, specificity: 73.9 %, AUC: 0.853; *p* < 0.05), followed by CRP (AUC: 0.863), IL-6 (AUC: 0.687), and fecal calprotectin (cutoff: 32.68 ng/g; sensitivity: 87.8 %, specificity: 76.7 %, AUC: 0.677; *p* < 0.05). For mortality prediction, fecal calprotectin showed the highest AUC (0.876) at a cutoff of 38.9 ng/g (sensitivity: 85.9 %, specificity: 75.1 %), followed by serum calprotectin (AUC: 0.820; *p* < 0.05), while CRP and IL-6 had lower AUC values (0.686 and 0.709 respectively) and were not statistically significant (Table 4).

**Table 4:** The predictive accuracy for surgical intervention and mortality.

	Surgical intervention					Mortality				
Variables	Cut-off	Sensitivity	Specificity	AUC	p	Cut-off	Sensitivity	Specificity	AUC	p
IL–6 (pg/mL)	25.75	78.9	68.6	0.687	0.148	25.65	85.7	65.3	0.709	0.082
CRP (mg/L)	7.9	79.4	77.8	0.863	**0.002**	10.1	85.7	69.8	0.686	0.121
Serum calprotectin (μg/mL)	37.84	88.9	73.9	0.853	**0.003**	49.78	88.7	78.1	0.82	**0.008**
Fecal calprotectin (μg/g)	32.68	87.8	76.7	0.677	**0.004**	38.9	85.9	75.1	0.876	**0.002**

## Discussion

Prediction and early diagnosis of NEC on an individual basis remains a challenge due to its complex and multifactorial nature. Although numerous observational studies have identified various clinical and non-clinical risk factors linked to the development of NEC, the prognostic significance of these factors is often uncertain. Prognostic research on NEC has predominantly focused on clinical parameters, but its ability to accurately predict outcomes is still limited.^15^

Fecal biomarkers, such as calprotectin^12^ human S100A12^16^ intestinal fatty acid-binding proteins (I-FABP)^17^ and intestinal alkaline phosphatase activity^18^ along with urinary biomarkers including I-FABP^17^ and serum amyloid A (SAA)^19^ and serum biomarkers such as cytosolic β-glucosidase^20^ SAA,^21^ inter-alpha inhibitor proteins^22^ various differentially expressed genes^23^ and absolute monocyte count, have all been investigated for their potential in the prediction and early detection of NEC. There are currently no studies that specifically evaluate serum calprotectin as a biomarker for NEC. However, its diagnostic potential has been explored in the context of neonatal sepsis.^24^

This pilot study supports the potential utility of serum and fecal calprotectin as prognostic biomarkers for assessing NEC severity and guiding surgical decision-making. The sensitivity and specificity values derived from ROC analysis for serum and fecal calprotectin suggest their strong predictive capabilities. Previous research has demonstrated the utility of serum calprotectin in inflammatory conditions, but its role in NEC remains less well-established. The current findings are in line with other studies that suggest the involvement of inflammatory mediators such as IL-6 and CRP in the pathogenesis and progression of NEC.

The ability to accurately predict mortality in NEC remains a critical challenge, as timely identification of high-risk infants could significantly improve clinical decision-making and outcomes. While markers like CRP have been traditionally used, their limited sensitivity in differentiating between survival outcomes underscores the need for more specific biomarkers. The present findings suggest that fecal calprotectin, given its higher sensitivity and specificity, may serve as a more accurate indicator for mortality risk, offering clinicians a valuable tool for stratifying patient risk and optimizing therapeutic interventions. Further research is essential to corroborate these results and explore calprotectin’s role within a broader prognostic framework for NEC.

Fecal calprotectin levels in neonates displayed a wide variability, ranging from 5.5 to 6000 μg/g, and showed no relationship with gestational age or birth weight. Meconium calprotectin levels were significantly higher than those measured after two weeks of life and were associated with both birth weight and the presence of meconium-stained amniotic fluid, while fecal calprotectin concentrations progressively decreased with postnatal age, showing greater reductions in breastfed infants compared to formula-fed infants by the third or fourth week of life [^14^]. The fecal calprotectin level in the control group was found to be 3.6 μg/g. The threshold values identified in the present study for fecal calprotectin are notably lower than those reported in previous studies. While the exact reason for this discrepancy is unclear, it may be influenced by differences in patient populations, study designs, or sample handling and analysis protocols. The present study used a specific ELISA kit and variability in assay methods could also contribute to differences in reported threshold values. Further multicenter studies are needed to standardize calprotectin measurement techniques and establish universally applicable thresholds.

In a study, stool calprotectin levels were elevated in 58 % of neonates diagnosed with NEC, compared to 13 % in those without the diagnosis. Furthermore, a cutoff value of 226 µg/g for fecal calprotectin was found to provide optimal diagnostic accuracy, with 75 % sensitivity and 76 % specificity in predicting NEC.^25^ In the present study, fecal calprotectin emerged as a strong predictor for surgical intervention, with a cutoff value of 32.68 μg/g, demonstrating high sensitivity and specificity. Additionally, fecal calprotectin at a cutoff of 38.9 μg/g was effective in predicting mortality, reinforcing its value as a marker for assessing NEC severity and related outcomes.

As a pilot study, the relatively small sample size is an inherent limitation and may reduce the statistical power to detect subtle differences, as well as limit the generalizability of the findings. However, the data provide valuable preliminary insights that can inform the design of larger, more definitive studies. Another important limitation is the use of a relatively small, randomly selected control group instead of matched controls based on clinical variables such as feeding type. The single-center setting may restrict external validity, as local practices or population characteristics may differ from those in other institutions. Additionally, unmeasured confounders such as nutritional factors, environmental exposures, and genetic variability may have influenced calprotectin levels. Lastly, variability in the timing of sample collection during the neonatal period could have affected biomarker concentrations, especially considering known fluctuations in fecal calprotectin associated with postnatal age and feeding type, such as breast milk versus formula.

## Conclusions

This pilot study provides preliminary insights into the potential roles of serum and fecal calprotectin as complementary biomarkers in the assessment of NEC severity and clinical outcomes. However, further research is needed to validate these findings, including larger, multicenter studies and the exploration of additional variables, to enhance the understanding of the combined diagnostic and prognostic value of serum and fecal calprotectin in neonates.

## Ethical approval

Ethical approval was obtained from the local ethics committee (number: 26,379,996/26, 03–2022), and informed consent was secured from the parents. This study adhered to relevant ethical standards and guidelines.

## Authors’ contributions

The study was conceptualized and designed by SE, CT, and IK, with data curation, formal analysis, and methodology contributions from SE, IK, and SK. Project administration was led by SE and CT, while investigation and supervision were carried out by SE, CT, SK, UC, and AK. Writing, including original draft preparation and review, was collaboratively performed by all authors.

## Funding

No funding was received for this study.

## Conflicts of interest

All authors declare no conflicts of interest, financial or personal relationships that could inappropriately influence or bias the results of this study.

## Data Availability

The data supporting the findings of this study were used under license and are not publicly available. However, the study protocol and a limited range of data are available from the corresponding author upon reasonable request.
